# What is important to people living with dementia?: the ‘long-list’ of outcome items in the development of a core outcome set for use in the evaluation of non-pharmacological community-based health and social care interventions

**DOI:** 10.1186/s12877-019-1103-5

**Published:** 2019-03-27

**Authors:** Andrew J. E. Harding, Hazel Morbey, Faraz Ahmed, Carol Opdebeeck, Reena Lasrado, Paula R. Williamson, Caroline Swarbrick, Iracema Leroi, David Challis, Ingrid Hellstrom, Alistair Burns, John Keady, Siobhan T. Reilly

**Affiliations:** 1 Faculty of Health and Medicine, Division of Health Research, Lancaster University, Lancaster, UK; 2 Department of Psychology, Manchester Metropolitan University, Manchester, UK; 3 Division of Nursing, Midwifery & Social Work, University of Manchester, Manchester, UK; 4 Clinical Trials Research Centre, University of Liverpool, Liverpool, UK; 5 Medical Research Council North West Hub for Trials Methodology Research, Liverpool, UK; 6 Division of Neuroscience & Experimental Psychology, University of Manchester, Manchester, UK; 7 Division of Population Health, Health Services Research & Primary Care, University of Manchester, Manchester, UK; 8 Department of Social and Welfare Studies, Linköping University, Linköping, Sweden; 9 Greater Manchester Mental Health NHS Foundation Trust, Manchester, UK

**Keywords:** Core outcome set, Delphi survey long-list, Dementia, Neighbourhood, Non-pharmacological, Literature review, Qualitative research

## Abstract

**Background:**

Core outcome sets (COS) prioritise outcomes based on their importance to key stakeholders, reduce reporting bias and increase comparability across studies. The first phase of a COS study is to form a ‘long-list’ of outcomes. Key stakeholders then decide on their importance. COS reporting is described as suboptimal and this first phase is often under-reported. Our objective was to develop a ‘long-list’ of outcome items for non-pharmacological interventions for people with dementia living at home.

**Methods:**

Three iterative phases were conducted. First, people living with dementia, care partners, health and social care professionals, policymakers and researchers (*n* = 55) took part in interviews or focus groups and were asked which outcomes were important. Second, existing dementia trials were identified from the ALOIS database. 248 of 1009 pharmacological studies met the inclusion criteria. Primary and secondary outcomes were extracted from a 50% random sample (*n* = 124) along with eight key reviews/qualitative papers and 38 policy documents. Third, extracted outcome items were translated onto an existing qualitative framework and mapped into domains. The research team removed areas of duplication and refined the ‘long-list’ in eight workshops.

**Results:**

One hundred seventy outcome items were extracted from the qualitative data and literature. The 170 outcome items were consolidated to 54 in four domains (Self-Managing Dementia Symptoms, Quality of Life, Friendly Neighbourhood & Home, Independence).

**Conclusions:**

This paper presents a transparent blueprint for ‘long-list’ development. Though a useful resource in their own right, the 54 outcome items will be distilled further in a modified Delphi survey and consensus meeting to identify core outcomes.

## Background

Recent estimates suggests that there are 850,000 people in the UK and 46.8 million globally who live with dementia [1]. Many people with dementia live alone and are particularly reliant on support in everyday living from family members, community services and home care agencies [2]. A range of different interventions are available for people living with dementia in their neighbourhoods and communities, including pharmacological and non-pharmacological (i.e. non drug based) interventions, to ensure support and care are offered throughout the person’s life. Support interventions at home, including environmental adaptations and assistive technologies, have been designed to enable people living with dementia to maintain their independence and live well with their diagnosis [3, 4]; however, evidence on the effectiveness of those interventions requires development [5, 6]. There is a growing demand to evaluate and improve the effectiveness of non-pharmacological community-based interventions and optimise outcomes for people living with dementia at home in their neighbourhoods and communities [7–9].

Robustly designed evaluations are necessary to determine if an intervention is effective. When looking at the effectiveness of an intervention(s), randomised experiments or trials are considered to be the ‘gold standard’ of evaluation [10]. One of the central pillars of evaluation and trial methodology is to compare outcomes of those receiving the experimental intervention and those who do not to measure and contrast beneficial or harmful effects. Ruling out almost all rival explanations for the outcomes observed is possible in a randomised experimental design. The selection of appropriate outcomes is therefore a crucial part of designing trials and evaluations [11, 12]. The term ‘outcome’ commonly refers to the impact of activity or support and services, and is defined in the Good Indicator Guide as “a measurable change in health status, sometimes attributable to a risk factor or an intervention” [13]. In practice, every intervention and/or service is designed to achieve an outcome. These outcomes can be specific or diverse focusing on, for example, quality of care, service supports, improvement in physical, mental or emotional wellbeing and so on [14].

However, existing outcome measures in relation to trials of non-pharmacological interventions for people living with dementia have two major limitations. The first limitation relates to which outcome measures are adopted and how choices are made to include outcome measures. While the choice of applied outcome(s) may be relevant to the intervention, what is important to those with lived experience of health conditions often contrasts with professional perspectives [15]. Indeed, outcomes that are salient to the intervention may not always be consistent with what people living with dementia value. This is perhaps symptomatic of a wider societal issue where people living with dementia are all too often positioned as a ‘seldom heard group’, encounter stigma on a regular basis[16], and are often not consulted about what outcomes are important to measure and, by implication, what interventions therefore might be desirable [16, 17]. Whilst the perspectives of people living with dementia and carers on desirable outcomes of community care captured by Bamford and Bruce constitutes important research [18], it is nearly two decades old. In recent years recommendations relating to outcome measures for use in dementia trials has mainly come from professional groups [19]. People living with dementia are rarely, if at all, consulted [20]. There is wider evidence from research in other fields that patients or the public have identified outcomes that were not previously identified by trialists [21]. The merit of many trials on non-pharmacological community-based interventions for people living with dementia, and possibly the effectiveness of interventions, may be questionable if research focuses on outcomes that are not identified as being of importance to people living with dementia and other key stakeholders.

The second limitation is that given a lack of consensus and transparency about which outcomes are salient and important, many completed and on-going studies that evaluate interventions for people living with dementia have a high degree of variation in relation to what outcomes are used and even how they are measured [8, 22]. This hinders the potential to compare for effectiveness across studies and makes both the meta-analysis and interpretation of results difficult.

These limitations provide the rationale for the creation of a core outcome set (COS) [11, 12]. The Core Outcome Measures in Effectiveness Trials (COMET) Initiative website (www.comet-initiative.org/) describes a COS as representing:

“…the minimum that should be measured and reported in all clinical trials... The existence or use of a core outcome set does not imply that outcomes in a particular trial should be restricted to those in the relevant core outcome set. Rather, there is an expectation that the core outcomes will be collected and reported, making it easier for the results of trials to be compared, contrasted and combined as appropriate; while researchers continue to explore other outcomes as well.”

In recent years, core outcome set developers have used a number of similar phases when creating a COS [11]. The first phase focuses on developing a comprehensive ‘long-list’ of outcomes that are currently used in existing trials and literature, along with outcomes that are identified as important through qualitative research with key stakeholder groups. Subsequent phases involve Delphi surveys to rate the importance of the identified ‘long-list’ of outcomes, a consensus meeting in order to arrive at agreement about what outcomes should be regarded as core, and a systematic review(s) and/or other methods to identify and assess existing measurement instruments that correspond to core outcomes [11].

This study methodology, including how we have utilised the lived experience of people living with dementia as co-researchers [23] to modify and design an accessible Delphi method is outlined in greater detail elsewhere [19, 24]. The clarity and transparency of COS reporting is described in a recent review as suboptimal [25] and the important initial phase of developing the ‘long-list’ often goes under reported. In this paper we report on the development of the ‘long-list’ of outcomes for use in non-pharmacological community based interventions for people with dementia who live at home.

## Methods

There were three parts within the first phase of this mixed method study. First (*Phase 1a)*, interviews and focus groups were undertaken with key stakeholder groups that asked which outcomes they thought were important from the perspectives of a person living with dementia. As outlined in the study protocol [19], these groups were:

1. *People living with dementia* - a person either formally or self-diagnosed with dementia and who lives at home.
2. *Care partners* [19] - a person with current or past experiences of providing care for a person living with dementia.
3. *Health and social care professionals* – a person currently or recently employed by a public or private organisation that provides care or support in a health or social setting for people living with dementia.
4. *Researchers* – a person with current or recent experiences of undertaking dementia related research (i.e. as denoted by being a lead or co-author on dementia related peer-reviewed publications, or involvement in current dementia-related research).
5. *Policymakers* - a person in a senior role with influence to shape national, regional or local dementia policy, or commission dementia service provision. This includes those who plan services.

Details of inclusion/exclusion criteria and recruitment processes can be found in the study protocol [19]. The second part of phase one (*Phase 1b)* relates to identifying outcomes as described in existing research literature. The third part of phase 1 (*Phase 1c)* relates to removing unsuitable items and consolidating outcomes extracted during 1a and 1b to a quantity that is manageable for the Delphi survey. The methods of each of these three parts are outlined below. For the purposes of this paper we are describing items identified as outcome items, but note that further work may be required to translate these outcome items into measurable outcomes in later stages of this study.

### Phase 1a: qualitative research with key stakeholders

Fifty-five people participated in 35 interviews and four focus groups. Participants included people living with dementia, care partners, health and social care professionals and policymakers. Participants were based across the UK, although mostly in the North West of England. Table 1 presents the participants and method.

**Table 1:** Participants in phase 1a qualitative research

Participant groups	Interviews	Focus groups	Total Participants
People living with dementia	14	1 (*n* = 3)	17
Care partners	8	2 (*n* = 5,5)	18
Health & social care professionals	8	1 (*n* = 7)	15
Policy makers	4	0	4
Researchers	1	0	1
	35	20	55

All interviews and focus groups were audio recorded, transcribed and data were managed in NVIVO version 11. The a-priori aim of the interviews and focus groups was to develop a list of outcomes identified as important by stakeholders. With this aim in mind, an adapted version of Braun and Clarke’s [26] six stages of thematic analysis was applied to develop a thematic framework. We outline here Braun and Clarke’s six stages and detail how we adapted them:

- Phase 1: Familiarisation: repeated reading of the data and reading the data in an active way.
- Phase 2: Generating initial codes: identifying emerging codes that relate to outcome items and areas that were considered to be important to people living with dementia.
- Phase 3: Searching for themes, looking through codes to see how they fit with each other.
- Phase 4: Themes were reviewed, including merging and collating them into groups where necessary, including use of a-priori themes adapted from Bamford and Bruce’s [18] framework to refine the themes further.
- Phase 5: Themes and outcome items were defined and names finalised.
- Phase 6: Given that we were developing a list of outcome items for a Delphi survey we did not produce an emerging narrative. Instead we developed a framework of outcome items deemed important to people living with dementia. This is the key adaption to Braun and Clarke’s [26] thematic analysis.

### Phase 1b: extraction of outcomes from existing trials

The Cochrane Dementia and Cognitive Improvement Group, at The Medical Sciences Division at Oxford University, manages the ALOIS database - a comprehensive and open access database of dementia studies (http://www.medicine.ox.ac.uk/alois/). ALOIS is updated by monthly searches of Medline, Embase, PsycINFO, Web of Science Core Collection, ClinicalTrials.gov, World Health Organisation International Clinical Trial Registry Platform meta-registry, Cochrane Controlled Register of Controlled Trials (CENTRAL) and LILACs (via Bireme) input into ALOIS. The database contains records of randomised controlled trials, controlled clinical trials and other ‘open-label’ studies. The advanced search function allows users to search by study aim, study design, intervention type (Any, Non-pharmacological, Pharmacological, Unclear or Both) or whether records are Cochrane studies. In January 2016 the ALOIS database was used to extract outcomes adopted in existing non-pharmacological interventions, applying the following inclusion criteria:

*Types of participants*

- People with dementia living at home in their neighbourhood/community

*Types of interventions*

Any UK based or international non-pharmacological intervention focusing on people living with dementia at home, which aimed to support people living with dementia in their neighbourhoods and communities. This included for example assistive technology (e.g. trials investigating the efficacy/outcomes associated with cognitive aids, environmental sensors, video and audio technologies, and advanced integrated sensor systems used in the home for people with dementia); psycho-social (e.g. psychodynamic approaches, reminiscence and life review therapy, support groups, reality orientation, memory training, and cognitive/behavioural approaches); psychological; social; nutritional (excluding medical supplements); educational; literature-based (e.g. book clubs); carer focused interventions if outcomes for people living with dementia were reported.

The following exclusion criteria were applied:

*Types of participants*

- People without a diagnosis of dementia (e.g. healthy older people, people with mild cognitive impairment)
- People living with dementia in any form of residential care

*Type of intervention*

- Pharmacological
- Electrophysiological
- Other medical device driven interventions

A total of 1009 studies registered as non-pharmacological were extracted from the ALOIS database, of which 248 studies met the inclusion criteria. The purpose of the literature review was to determine the outcomes used in existing research to complement the outcomes identified from interviews and focus groups with people with dementia, their care partners, health and social care professionals, and policymakers, commissioners and research leaders. As such, we extracted outcomes from a 50% random sample of the identified studies (*n*=124). During the outcomes extraction process we observed that no new outcome was added and that we had reached a saturation point at 50%. Outcomes and their respective measures or tools used were extracted verbatim into a MS Excel spreadsheet. In addition, and in order to be as comprehensive as possible, key reviews and qualitative studies (*n* = 8) and policy documents (*n* = 38) were also identified based on the knowledge of the research team and the study advisory group. All outcomes from these 170 sources were extracted and added to the aforementioned framework, if not already included, to constitute a ‘long-list’ of outcome items.

### Phase 1c: developing the ‘long-list’: researcher/clinician group based workshops

In an approach similar to that of other COS developers [27], eight sequential workshops with members of the research team were convened with a view to remove duplication, further consolidate areas of commonality and map outcome items into domains. These workshops were adapted from existing interactive focus group approaches [28, 29]. Each workshop typically involved 4–6 people. All meetings where attended by AH, HM, FA and SR. IL, JK and DC independently attended one workshop each. CS and PW provided input over email. Each workshop was between 2 and 4 h in duration. Advisory group members were also sent iterations of outcome item frameworks and provided input at different stages.

Participants were sent copies of the existing framework of outcome items in preparation for each workshop. Sending these materials in advance allowed participants to become familiar with the current configuration of outcome items and to form points of discussion about how and why outcome items should be moved or removed. In the workshop, each outcome item was listed in a spreadsheet and printed on individual pieces of paper. These were subsequently laid out on large tables and participants engaged in discussion about each. Individual outcome items were reviewed and re/positioned under different domains while participants exchanged views around the rationale for mapping outcome items into domains, merging or removing them. Decisions were recorded and logged following each workshop allowing the research team to track the journey of outcome items that had been moved or removed and also to revisit the rationale for decisions taken. The team met until there was consensus that this deliberative analysis had reached saturation.

## Results

A total of 170 items were extracted from the qualitative data and existing literature. The origin of these 170 items are as follows:

- 79 were extracted from qualitative data collection with key stakeholders
- 77 were extracted from the reviewed non-pharmacological studies registered on the ALOIS database
- 13 were extracted from policy documents
- One additional area was extracted from key reviews and qualitative papers.

However, as outlined in the previous section where possible outcomes extracted from existing studies were added to the existing qualitative framework. On this basis, during the initial extraction, forty-nine of the outcomes extracted from the literature were able to be mapped on to the qualitative framework, establishing 121 outcome items – of which 28 originated from the literature. The outcome items were placed in initial groupings by the research team upon extraction. These 121 outcome items, and the actions taken over the eight workshops (i.e. whether they were merged or removed) for each item, is presented in Table 2.

**Table 2:** One hundred twenty-one outcomes extracted from qualitative data collection, across literature and their final status

Source	No.	Initial grouping	Outcome	Final status
Qualitative data collection (1–79)		Living with dementia		
	1		*Acceptance of dementia*	In long-list
	2		*Access to meaningful activity and stimulation*	In long-list
	3		*Access to social contact and company*	In long-list
	4		*Being personally clean and comfortable*	In long-list
	5		*Choice in living arrangements*	Removed (not relevant)
	6		*Feeling financially secure*	In long-list
	7		*Feeling safe and secure*	In long-list
	8		*Having a sense of social integration*	In long-list
	9		*Living in a clean and comfortable environment*	Merged (4)
	10		*Maintaining a sense of self*	In long-list
	11		*Maintaining a sense of who you are, role/occupation*	In long-list
	12		*Self-esteem*	In long-list
	13		*Having a sense of purpose*	Merged (11)
	14		*Religion/spirituality*	Merged (2)
	15		*Basic Activities - being able to carry out basic self-care and basic tasks*	In long-list
	16		*Instrumental/more complex activities*	In long-list
		Maintaining cognition		
	17		*Word finding/language difficulties*	In long-list
	18		*Difficulties identifying/counting money/overspending*	In long-list
	19		*Deterioration (fears it will ‘spread’)*	In long-list
	20		*Memory*	In long-list as 2 items (short & long term memory)
	21		*Visuospatial abilities*	In long-list
	22		*Confusion/getting lost*	In long-list
	23		*Learning new things*	In long-list
		Physical functioning		
	24		*Physical function*	In long-list
	25		*Maintaining physical function*	Merged (27)
	26		*Falls*	In long-list
	27		*Keeping physically active*	In long-list
	28		*Balance*	In long-list
	29		*Co-morbidity*	In long-list
	30		*Mobility*	In long-list
		Maintaining relationships		
	31		*Importance of relationships with family and friends*	In long-list
	32		*Loss of relationships*	Merged (31)
	33		*Reaction of family and friends to diagnosis*	In long-list
		Maximising autonomy or independence		
	34		*Maintaining independence*	Became a domain - Independence
	35		*Being able to make choices/being involved in choices*	Became a domain - Independence
	36		*Maximising confidence*	Merged (12)
	37		*Minimising loneliness or isolation*	In long-list
		Living with others and the environment		
	38		*Access to appropriate services*	Removed (Intermediate/service/process outcome)
	39		*Information,* i.e. *having information about available services/support*	Removed (Intermediate/service/process outcome)
	40		*Being able to relate to other service users*	Merged (3)
	41		*Being treated as an individual*	Removed (Intermediate/service/process outcome)
	42		*Communication*	In long-list
	43		*Continuity of hobbies and care*	Merged (2)
	44		*Feeling valued and respected by others*	In long-list
	45		*Having a safe and secure home*	Merged (7)
	46		*Having a safe and secure neighbourhood*	In long-list
	47		*Having a say in services*	Merged (41)
	48		*Neighbourhood and public awareness of dementia*	Became a domain - Friendly Neighbourhood & Home
	49		*Service driven provision*	Removed (process outcome)
		Psychological and behavioural symptoms of dementia (BPSD)		
		Behaviour		
	50		*Frustration*	In long-list
	51		*Sleeping*	In long-list
	52		*Denial*	Merged (1)
	53		*Aggression/abusive behaviour*	In long-list
	54		*Agitation*	In long-list
	55		*Apathy*	In long-list
	56		*Suspicion/paranoia*	In long-list
	57		*Changes in personality*	Removed (present across BPSD outcomes)
	58		*Eating*	In long-list
	59		*Wandering*	In long-list
	60		*Inappropriate speech/disinhibition*	In long-list
	61		*Repeated questioning*	In long-list
	62		*Hallucinations/delusions*	In long-list
		Mood		
	63		*Anxiety*	In long-list
	64		*Embarrassment*	In long-list
	65		*Happy*	In long-list
	66		*Depression*	In long-list
		The experience of caring for a person with dementia		
	67		*The effect of caregiving on the carer*	Removed (not relevant - focus not on person living with dementia)
	68		*Carer reaction*	Removed (not relevant - focus not on person living with dementia)
		Others		
	69		*Quality of life*	Became a domain - Quality of Life
	70		*Lived experience*	Removed (present across many outcomes)
	71		*Health and co-morbidity*	In long-list
	72		*End of life planning*	Removed (Intermediate/service/process outcome)
	73		*Cultural differences*	Merged (41)
	74		*Cost effectiveness of interventions*	Removed (Intermediate/service/process outcome)
	75		*Managing dementia*	Became a domain - ‘Self-Managing Dementia Symptoms’
	76		*Dementia progression*	Merged (19)
	77		*Medical events/falls*	Merged (26)
	78		*Maintaining everyday activities*	Merged (2, 15, 16)
	79		*Support*	Merged (2, 3, 31, 38)
Additional outcomes from literature (80–107)	80		*Adverse events*	Removed (not relevant)
	81		*Aberrant behaviours other than wandering*	Merged (59)
	82		*Secretiveness*	In long-list
	83		*Hopelessness*	Merged (66)
	84		*Subjective memory/cognitive problems*	Merged (17–23)
	85		*General cognition*	Became a domain - ‘Self-Managing Dementia Symptoms’
	86		*Alertness*	In long-list
	87		*Attachment*	Merged (31–32)
	88		*Personal cost to person with dementia/family*	Removed (process outcome/cost)
	89		*Mortality*	Removed (not relevant)
	90		*Time to significant event*	Removed (Intermediate/service/process outcome)
	91		*Physical environment*	Merged (part of ‘Friendly Neighbourhoods & Home’ domain)
	92		*Medications (Type; Access; management)*	Merged (15, 16)
	93		*Medical health markers (cardiac rhythm, brain activation, brain volume, blood pressure)*	Merged (29, 71)
	94		*Number of contacts with health and social care professionals*	Removed (Intermediate/service/process outcome)
	95		*Health Behaviours*	Removed (Intermediate/service/process outcome)
	96		*Weight (also BMI)*	Merged (93)
	97		*Sleep*	In long-list
	98		*Quality of care process outcomes*	Removed (Intermediate/service/process outcome)
	99		*Health related quality of life*	Merged (Quality of Life domain and present across outcomes)
	100		*Enjoyment*	Merged (present across ‘Friendly Neighbourhoods & Home’ domain)
	101		*Self-efficacy*	Merged (12)
	102		*Wellbeing*	Removed (present across included outcomes)
	103		*Use of healthcare*	Removed (cost related, not individual outcome)
	104		*Use of social resources*	Removed (cost related, not individual outcome)
	105		*Use of other organisations*	Removed (cost related, not individual outcome)
	106		*Unmet needs general*	Removed (not a single outcome)
	107		*Satisfaction with/acceptability of intervention*	Removed (Intermediate/service/process outcome)
Additional outcomes from policy (108–120)	108		*Timely diagnosis*	Removed (Intermediate/service/process outcome)
	109		*Stigma or discrimination*	Merged (44)
	110		*Sense of humour*	Merged (114)
	111		*Research*	Removed (not an outcome)
	112		*Reaction of others or wider community*	Merged (48)
	113		*Impact of diagnosis*	Merged (present across all included outcomes)
	114		*Having a laugh*	In long-list
	115		*Getting out of the house*	Merged (2, 34, 36)
	116		*Feeling like a burden*	In long-list
	117		*Faith religion or spirituality*	Merged (2)
	118		*Enjoyment*	Merged (100)
	119		*Empower or protection of rights*	Removed (Intermediate/service/process outcome)
	120		*Dementia friendly environments*	Merged (48)
Additional outcomes from key reviews and qualitative papers	121		*Missing something*	Merged (2, 78)
Added during team workshops	122		*Vision and hearing*	In long-list

The eight workshops previously described reduced the 121 outcome items to 53 and categorised them into domains. Some outcome items were selected as overarching domains, others were merged or removed. Outcome items were merged if they were judged sufficiently similar to other items, or even present across a cluster or number of outcome items. A common reason for removing an outcome item was if it fell outside of the remit of the COS. This included that the item did not focus on the person living with dementia, was a cost related outcome or was an intermediate/service/process outcome. One outcome item was removed because it referred to wider structural factors around the locus of care (choice in living arrangements). Two further outcome items, adverse events and mortality, were removed because they were associated with clinical interventions and subsequently were judged inappropriate for a core outcome set measuring individual outcomes in non-pharmacological health and social care interventions located in the community.

**Fig. 1:**
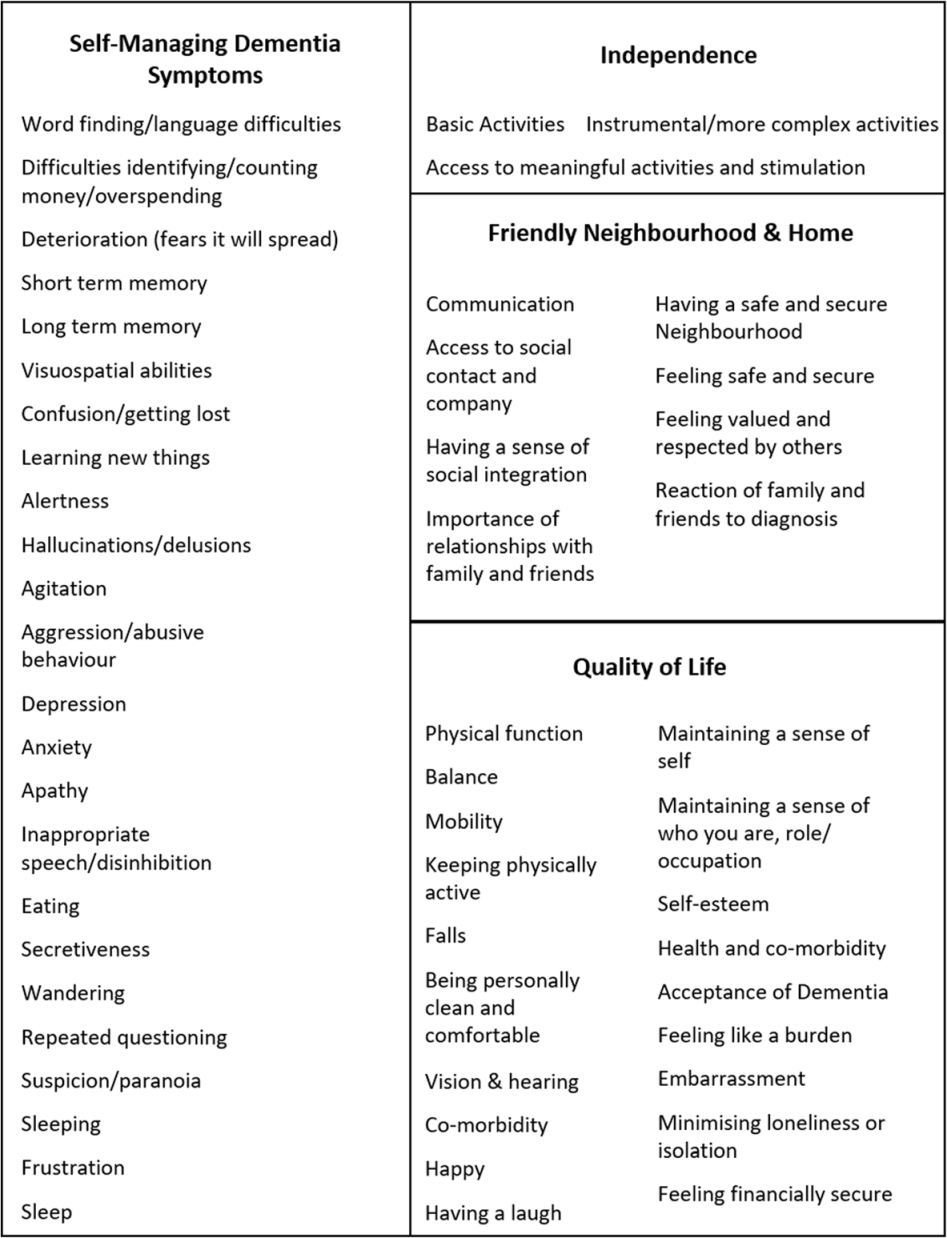
Fifty-four unique outcomes identified in qualitative research with key stakeholders and existing literature

Sixty-eight outcome items were either merged or removed, and one outcome item was added (Vision & hearing) during the workshops. The 54 outcome items were organised under four domains: Self-Managing Dementia Symptoms; Quality of Life; Friendly Neighbourhood & Home; Independence Fig. 1.

These 54 outcome items in the ‘long-list’ were formed into an accessible Delphi survey. The process of developing the ‘long-list’ into an accessible Delphi survey involved people living with dementia in their capacity as co-researchers, and will be and is reported on and discussed elsewhere [24].

## Discussion

The methods presented in this paper provide a comprehensive account of the identification of 54 outcome items of importance to key stakeholders, including people living with dementia, and that are used in existing trials of non-pharmacological interventions or found in other key literature.

The status of the perspectives of people living with dementia was a key consideration from the beginning of this study to address critical limitations that are outlined earlier. The privileging of people living with dementia’s perspective is evident in the manner the ‘long-list’ was formed. For example, outcomes in existing trials were mapped onto the qualitative framework that was initially drawn from Bamford and Bruce’s [18] work that promoted perspectives of people living with dementia and was expanded with outcome items extracted from qualitative data collection. Subsequently, many outcomes found in trials were mapped on to the qualitative framework. Furthermore, a key part of designing an accessible Delphi survey was utilising the lived experience of people living with dementia as co-researchers in the study design [19]. The privileging of the views of people living with dementia who participated in the study when forming the ‘long-list’, and their role as co-researchers in the Delphi design (as reported elsewhere [24]), will ensure that the final core outcome set reflects what is important to those with lived experience.

A further illustration of privileging the views of people living with dementia is in the qualitative data collection. Other stakeholder groups were asked to respond in interviews and focus groups through empathic reflection whereby they considered important outcome items *from the perspective of a person living with dementia*, as opposed to what they thought was important from their own perspective. There is clearly a distinction between what a carer or healthcare professional would consider important, and what they think a person living with dementia might deem important. While we recognise there will be convergence of perspectives to some extent when a stakeholder reflects in this way, we recognise the intersubjective nature of multiple perspectives of complex experiences gathered through qualitative data collection methodology [30].

This approach is likely to have produced a different configuration and description of outcome items when compared to existing outcome measurement instruments. More specifically, the ‘long-list’ of outcome items in this paper may present specific individual items that are part of or regarded as a construct of an existing outcome or outcome measure, for which single composite scores tend to be presented. An example is how the individual items and constructs in the neuropsychiatric inventory [31] are present in the ‘long-list’ as individual outcome items. This is the consequence of the methods undertaken - the interviews and focus groups - in attaining granularity of what outcome items are regarded as important. We suggest this is a key strength of the work considering the ‘long-list’ reflects what is valued as important by people living with dementia and other key stakeholders.

Given the participants in the qualitative data were based in the UK the ‘long-list’ has particular relevance for the UK context as well as being of international interest. That said, although the trials reviewed were international, more work is needed to ensure that the outcome items are as equally applicable for an international context. It is important to note that the outcome items in the ‘long-list’ are not salient to any one type of intervention. The ‘long-list’ may benefit practitioners, service planners and commissioners as it serves to direct the focus of health and social care services and interventions having been derived through robust methods and informed by people living with dementia and carer partners. On this basis, the 54 outcome items presented in this paper acts as a useful resource or an ‘outcome items bank’ for researchers, service providers and commissioners alike. The ‘long-list’ reported here does not include outcomes relating to care partners, delivery of care or processes, costs or economic related outcomes. Research focusing on care partner related outcomes is warranted as a separate study and clearly an area of interest for further research.

## Conclusions

In COS research that uses a Delphi approach, if a large quantity of outcome items are found it will often not be manageable or realistic to include every outcome item extracted from qualitative research methods and literature in a Delphi survey. This paper outlines a transparent blueprint around how a ‘long-list’ of outcome items was formed through a process of extraction from consultation with key stakeholder groups. In addition, this paper presents a robust approach to identify duplication, merge areas and remove unsuitable outcome items. More broadly, the methods and ‘long-list’ outlined here are capable of informing wider health and social care studies where there is a focus on priority setting and the inclusion of areas of importance to people living with dementia.

The 54 outcome items in the ‘long-list’ will be distilled further through a modified Delphi survey with key stakeholders and a consensus meeting, both including people living with dementia, where core outcomes will be identified [19]. In a further phase we will review existing outcome measurement instruments in order to highlight how (if at all) measurement instruments measure the core outcomes identified.

## Abbreviations

COMET: Core Outcome Measures in Effectiveness Trials
COS: Core outcome set
